# TDP-43 Toxicity in Yeast Is Associated with a Reduction in Autophagy, and Deletions of *TIP41* and *PBP1* Counteract These Effects

**DOI:** 10.3390/v14102264

**Published:** 2022-10-15

**Authors:** Sei-Kyoung Park, Sangeun Park, Susan W. Liebman

**Affiliations:** Department of Pharmacology, University of Nevada, Reno, NV 89557, USA

**Keywords:** TIP41, TDP-43, PBP1, yeast, autophagy

## Abstract

When human TDP-43 is overexpressed in yeast it is toxic and forms cytoplasmic aggregates. The mechanism of this toxicity is unknown. Genetic screens for TDP-43 toxicity modifiers in the yeast system previously identified proteins, including PBP1, that enhance TDP-43 toxicity. The determination in yeast that deletion of *PBP1* reduces TDP-43 toxicity while overexpression enhances toxicity, led to the discovery that its human homolog, *ATXN2*, is associated with ALS risk. Thus, the yeast system has relevance to human disease. We now show that deletion of a new yeast gene, *tip41Δ*, likewise suppresses TDP-43 toxicity. We also found that TDP-43 overexpression and toxicity is associated with reduced autophagy. This is consistent with findings in other systems that increasing autophagy reduces TDP-43 toxicity and is in contrast to a report of enhanced autophagy when TDP-43 was overexpressed in yeast. Interestingly, we found that deletions of *PBP1* and *TIP41*, which reduced TDP-43 toxicity, eliminated TDP-43′s inhibition of autophagy. This suggests that toxicity of TDP-43 expressed in yeast is in part due to its inhibition of autophagy and that deletions of *PBP1* and *TIP41* may reduce TDP-43 toxicity by preventing TDP-43 from inhibiting autophagy.

## 1. Introduction 

Yeast has been useful in the study of human proteins that form amyloid or prion-like aggregates associated with certain diseases [1,2,3,4,5,6,7]. When wild-type human TDP-43, which frequently aggregates in sporadic ALS [8], is overexpressed in yeast, it is toxic and forms cytoplasmic aggregates [3]. Mutants in TDP-43 associated with familial ALS cause toxicity at a lower level of expression than does wild-type TDP-43 [8,9]. Since TDP-43 is not normally present in yeast, this model only looks at positive effects of TDP-43 on toxicity, although loss of TDP-43 function in higher cells is now also known to be pathogenic [10]. 

Genetic screens for modifiers of TDP-43 toxicity in the yeast system identified proteins involved in TDP-43 on toxicity [3,11,12,13,14,15]. Indeed, the finding that deletion or overexpression of *PBP1,* respectively reduces or enhances TDP-43 toxicity led to the discovery that *PBP1′s* human homolog, *ATXN2*, is associated with ALS risk. Research shows that an increase in the size of the polyglutamine expansions in *ATXN2,* within the normal range, is associated with enhanced risk for ALS [16,17]. Furthermore, reduced expression of *ATXN2* extends life and reduces toxicity in a model of TDP-43 proteinopathy in mice [18]. The ability of the yeast screen to identify a mammalian protein that effects ALS risk in humans and TDP-43 toxicity in mice indicates that the yeast model is relevant to human disease.

It is unknown how *PBP1/ATXN2* affects TDP-43 toxicity [19]. However, *PBP1* is one of five yeast genes (*CCT6, SSB1, ICY1, TIP41*, *PBP1*) with similar phenotypes: overexpression of any of the proteins they encode rescues yeast with non-functional mitochondria (“petites”) from death caused by inefficient mitochondrial import of proteins [20]. In addition, deletion of any of these genes is lethal in petites [20]. Because of their phenotypic similarity to *PBP1*, we asked if deletions of *SSB1, ICY1,* or *TIP41* likewise effect toxicity of TDP-43, excluding *CCT6* because it is essential. Our results show that that indeed, *tip41Δ,* like *pbp1Δ,* suppresses TDP-43 toxicity while no effects were seen for the other deletions. 

Recently Yang et al. [21] showed that overexpression of PBP1, or amino acid starvation, causes PBP1 to form a liquid-like gel when yeast are grown under conditions requiring respiration. They also show that this PBP1 gel inhibits TORC1 under these conditions, thereby turning on autophagy. 

Since TDP-43 was reported to increase autophagy in yeast [22], we wondered if the stress of TDP-43 toxicity, like amino acid starvation, induced PBP1 to form a gel and enhance autophagy. To test this, we planned to ask if deletion of *PBP1* or *TIP41* would reduce the enhanced autophagy associated with TDP-43 overexpression. If so, it might mean that enhanced autophagy is part of the cause of TDP-43 toxicity and reduction of TDP-43 toxicity by *pbp1Δ* or *tip41Δ* could be due to a lowering of autophagy. 

To our surprise we found TDP-43 overexpression and toxicity was associated with reduced, not enhanced, autophagy. This is consistent with experiments in other systems that show autophagy reduces TDP-43 toxicity [23,24] and TDP-43 aggregates inhibit autophagy [25,26,27,28]. Finally, we found that deletions of *PBP1* and *TIP41*, which reduced TDP-43 toxicity, also eliminated TDP-43′s inhibition of autophagy. 

## 2. Materials and Methods

Strains and Plasmids: Yeast strain BY4741 (L3270) ([*PIN*^+^] *MATa his3Δ1 leu2Δ0 met15Δ0 ura3Δ0*), and isogenic deletions from the yeast deletion library made in this strain, were obtained from Open Biosystems, Huntsville, AL; Cat # YSC1053 [29]. Plasmid p*GAL1-TDP-43* (alias p2368, pAG415 *GAL1-TDP-43, LEU2, CEN*) was used to overexpress TDP-43 [30]. Its vector control is pAG415 *GAL1-ccdB* (alias p2245, Addgene #14145 donated by A. Gitler). Plasmid phER (alias p798; pHCA/*GAL4(1–93) ER.VP16, HIS3, CEN* Addgene # 108216 donated by D. Picard) was used to induce expression of *GAL1* controlled TDP-43 on dextrose media upon the addition of 2 µM β-estradiol. phER contains the human estrogen receptor hormone-binding domain fused to the *GAL4* DNA binding domain and the VP16 viral transcriptional activator (hER). Plasmid *pCUP1-GFP-ATG8* (alias p2571 *URA3, CEN*, Addgene #49423 from D. Klionsky’s lab) was used to measure autophagy.

**Figure 1:**
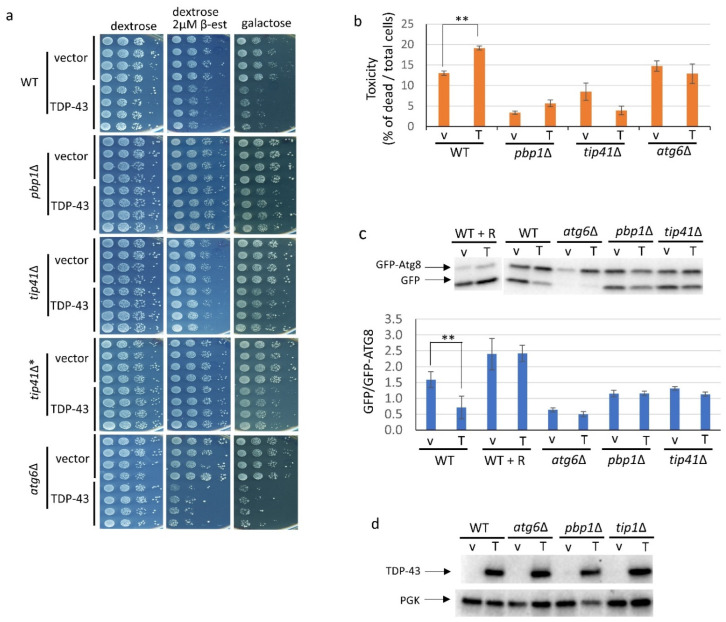
TIP41 is a new modifier of TDP-43 toxicity that reverses inhibition of growth and autophagy caused by TDP-43. BY4741 (WT), along with deletions of *pbp1∆, tip41∆* or *atg6∆* made in BY4741 were triple transformed with: p*GAL1-TDP-43* (TDP-43 or T) which enables overexpression of TDP-43 or its empty vector (vector or v), *pCUP1-GFP-ATG8* to measure autophagy, and phER which enables expression of *GAL1* controlled TDP-43 on dextrose media upon the addition of 2 µM β-estradiol. Addition of 50 µM Cu^++^, which induced GFP-ATG8, caused fluorescence but had no effect on cell morphology or growth rate. Expression of TDP-43 on galactose, or dextrose with β-estradiol, was confirmed by the presence of elongated cell shapes [13]. (**a**) Growth inhibitory effects due to TDP-43 overexpression are partially relieved by deletion of *PBP1* or *TIP41*. To examine the effects of TDP-43 on growth, four independent transformants of each type grown in plasmid selective media were normalized to 4 OD_600_, serially diluted 10-fold, and spotted on plasmid selective synthetic dextrose medium with and without 2 µM β-estradiol or on plasmid selective galactose medium supplemented with 1% raffinose. All media contained 50 µM Cu^++^. Plates were scanned after incubation at 30 °C for 3 days for glucose media and after 6–7 days for galactose media. The deletion strains were from the Open Biosystems yeast deletion library except for tip41∆* which is a new deletion remade in BY4741 for this study. (**b**) Cytotoxicity of the *GAL1* driven TDP-43 in WT, *pbp1*∆, *tip41*∆ and *atg6*∆ cells. Samples of cultures of triple transformants grown in plasmid selective 2% galactose with 2% raffinose liquid medium for 18 hrs to induce TDP-43 expression were stained with trypan blue and imaged. Raffinose was added to improve growth. The % of dead cells was calculated as the ratio of dead blue cells among total blue and white cells. Error bars present standard error of the mean (SEM) calculated from three independent transformants. (**c**) TDP-43 inhibits autophagy and *pbp1∆* or *tip41∆* reverse this effect. Autophagy is seen as the ratio of cleaved GFP to uncleaved GFP-ATG8 imaged with α-GFP (1:5000, F. Hoffmann-La Roche AG, Basil, Switzerland. Above is one representative gel. Below is average and SEM of an image J analysis of the ratio of the density of GFP to GFP-Atg8 bands in gels from three independent transformants. R indicates the addition of 200 nM rapamycin in plasmid selective TDP-43 and GFP-ATG8 overexpression media. ** indicates *p* < 0.05 using paired *t*-test. (**d**) The ability of *pbp1*∆, *tip41*∆, or *atg6*∆ to alter TDP-43 toxicity is not mediated by changing the cellular level of TDP-43. Immunoblot analysis established the level of TDP-43 in the transformants using α-TDP-43 (1:3000; Proteintech Group) and the level of the internal loading control, PGK1 using α-PGK (yeast 3-phosphoglycerate kinase, 1:10,000, Novex).

An independent *TIP41* deletion strain (shown as tip41Δ* on Figure 1a) was reconstructed in the same parent strain used for the deletion, BY4741. DNA from the deletion library strain with *kanMX4* replacing *TIP41* was amplified with the 45 uptag (TGACCTAAGGGCAGCTTTAGACACAACAGCTCCCCAGAAAAAATG) and downtag (GACGTGTATGTATTTGTACGTATTGTTTTGTATATTTGATTGTTA) primers surrounding the disruption. The amplicon was transformed into BY4741 and new *tip41Δ* *kanMX4* disruptions were selected on YPD supplemented with 200 mg/L geneticin (G418). The new presumptive *kanMX4* resistant *TIP41* deletion was confirmed by comparing its PCR products with PCR products from the *bona fide TIP41* deletion strain and a wild-type control using confirmation primers (A=CATGGCTTCTTTTGACTATCTGTTT; B=ATGATATTTAAGGAATCGTCGTTCA; C= GAATCCAAGGAATTTGAAGGTAAAT and D= TAGACATCTTGTACCAGTGAACGTG).

We constructed pGAL-TIP41 with Gateway cloning [31]. We made the *TIP41* entry clone with a BP reaction between pDONR221 and a PCR amplified *TIP41* fragment containing a stop codon. We made the pGAL1-TIP41 construct (p2683) with a LR reaction between the *TIP41* entry clone (p2676) and the yeast destination vector, pAG413 GAL-ccdB (Addgene #14141).

Scoring for growth: Cells were grown in standard yeast media [32]. To examine the effects of TDP-43 on growth, independent transformants of each type were suspended in sterile water, normalized to 4 OD_600_, serially diluted 10-fold, and spotted on plasmid selective synthetic dextrose medium with and without 2 µM β-estradiol or on plasmid selective galactose medium supplemented with 1% raffinose using a MC48 (Dan-kar Corp, MA) spotter (Figure 1a). Plates were scanned after incubation at 30 °C for 3 days for glucose media and after 6–7 days for galactose media.

Scoring for viability: Cells grown in liquid plasmid selective 2% galactose medium supplemented with 2% raffinose and 50 µM Cu^++^ (galactose) for 18 hrs at 30 °C with shaking were then resuspended in 1/10th volume TE and 1.5 μL were mixed on slides with 1.5 μL 0.4% trypan blue. The number of blue (dead) vs. unstained (viable) cells were counted blind in different fields of photographed cells imaged with a Nikon Eclipse E600 fluorescent microscope (100× oil immersion). Between 820 and 1106 cells were counted for each sample. 

Scoring for autophagy: Triple transformants with p*GAL1-TDP-43,* p*CUP1-GFP-ATG8,* and phER, and were selected on synthetic dextrose (2%) plates lacking leucine, uracil, and histidine (SD -Leu -Ura -His). To confirm that transformants could overexpress TDP-43 and express GFP-ATG8 these plates were replica-plated to SRGal (2% galactose) -Leu -Ura -His supplemented with 1% raffinose and 50 µM Cu^++^ where the *GAL1* and *CUP1* promoters were induced. Replica-plated colonies were checked for cell elongation to confirm overexpression of TDP-43 [12], and for GFP-ATG8 fluorescence. These colonies were used to determine either the amount of GFP-ATG8 cleavage (measured by immunoblotting) or the cellular location of GFP (measured by microscopic evaluation as described below) to report on the level of autophagy.

To determine the level of cleavage of GFP-ATG8 cells were grown in plasmid selective glucose media (SD -Leu -Ura -His), harvested, washed, normalized to 0.2 OD_600_ and spread on TDP-43 and GFP-ATG8 overexpressing plasmid selective galactose media (SRGal -Leu- Ura -His) with 1% raffinose and 50 µM CuSO_4_ without or with of 200 nM rapamycin [33]. Cells were washed off plates after overnight incubation (18 hrs) at 30 °C and resuspended in 250 µL of lysis buffer (80 mM Tris, 300 mM KCl, 10 mM MgCl2 and 20% glycerol), 1:50 diluted protease inhibitor cocktail (Sigma), and 5 mM phenylmethylsulfonylfluoride (PMSF) at pH 7.6. Cells were then lysed with 0.5 mm glass beads in a Biospec mini-beater 5X at high speed for 45 s each, separated by 1 min cooling in ice. Lysates cleared by centrifugation at 10,000× *g* for 5 min at 4 °C, were normalized so that 55 µg of total protein would be loaded on the gel lane, boiled for 5 min in sample buffer [4% SDS, 10% glycerol, 50 mM Tris-Cl pH6.8, 1 mM EDTA, 80 mM DTT, 0.05% (*w*/*v*) bromophenyl blue dye], and resolved on 10% SDS-PAGE followed by immunoblotting with α-GFP (1:5000, Roche) to compare the level of uncleaved GFP-ATG8 and cleaved GFP, with α-TDP-43 (1:3000; Proteintech Group) to measure the level of TDP-43, and with α-Pgk (yeast 3-phosphoglycerate kinase, 1:10,000, Novex) for an internal loading control. The ratio of cleaved GFP to uncleaved GFP-ATG8 measures autophagy. To compare the level of TDP-43 present in WT vs. deletion strains the internal PGK control was used. 

The cellular location of GFP-ATG8 determined by microscopy was also used to measure autophagy. Triple transformants verified in Figure 1a were grown in 2% dextrose plasmid selective media, normalized, reinoculated at 0.02 OD_600_ into 2% galactose medium supplemented with 2% raffinose, 50 µM Cu^++^ and 1.6 nM FM4-64 (Sigma, T3166, dissolved in DMSO) and grown for 18 hrs with shaking at 30 °C to visualize cell membranes and vacuoles. Fluorescence was visualized with a Nikon Eclipse E600 fluorescent microscope (100× oil immersion) equipped with GFP and mCh filters. Cells were examined for GFP-ATG8 fluorescence, FM4-64 staining, and trypan blue staining. Cells with diffuse fluorescence in the cytoplasm without a concentration of fluorescence in the vacuole, or fluorescence in cytoplasmic foci even if accumulation of fluorescence in the vacuole, were scored as exhibiting inhibited autophagy. Cells with a concentration of fluorescence in the vacuole without punctate cytoplasmic foci, or live cells with no fluorescence, were scored as having undergone autophagy. Dead cells were detected with trypan blue staining seen in bright field.

## 3. Results

### 3.1. Deletion of TIP41 Reduces Toxicity of TDP-43

Initial screening showed that deletions of *ICY1* and *SSB1* did not affect toxicity associated with galactose induced overexpression of TDP43, while deletion of *TIP41* had a big effect. Thus, we focused on *TIP41*. The effect of overexpression of TDP-43 on growth and cell viability was compared in yeast strain BY4741 and isogenic deletions of *TIP41*, *PBP1 and ATG6* made in that strain background. These three strains were transformed with *pGAL-TDP-43* (or its empty vector control) to allow for overexpression of TDP-43 on galactose, phER to allow the addition of 2 µM β-estradiol to induce overexpression of TDP-43 from *pGAL-TDP-43* in dextrose media, and *pCUP1-GFP-ATG8* to express GFP-ATG8 in the presence of 50 µM Cu^++^ to enable measurement of autophagy. Growth of spots of serial dilutions of at least four independent transformants was compared on plates and the fraction of dead cells was determined in liquid cultures (Figure 1a,b). As seen previously [16], overexpression of TDP-43 caused reduced growth and cell viability and these effects were ameliorated by deletion of *PBP1*. We found that deletion of *TIP41* likewise reduced these toxic effects of TDP-43 overexpression (Figure 1a,b). This finding was confirmed by using an independently made deletion of *TIP41* (Figure 1a). The effects of *pbp1Δ* and *tip41Δ* on TDP-43 toxicity were not caused by reducing the level of TDP-43 in the cell (Figure 1d).

### 3.2. Overexpression of TDP-43 Reduces Autophagy but Only in the Presence of PBP1 and TIP41

**Figure 2:**
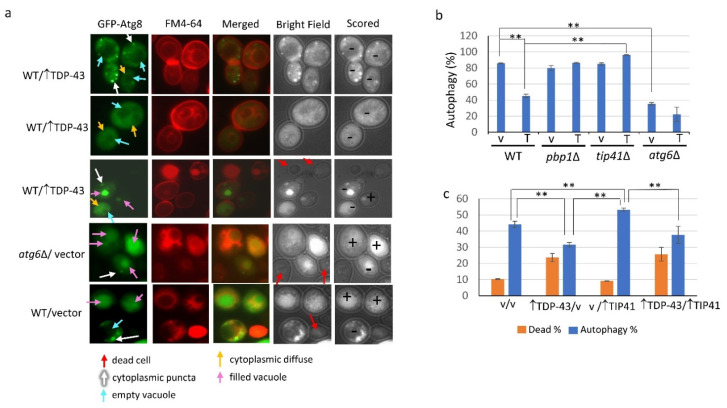
Microscopic assay showing TDP-43 overexpression reduces autophagy. Triple transformants analyzed in Figure 1a were inoculated at 0.02 OD_600_ in the presence of 1.6 nM FM4-64 to visualize cell membranes and vacuoles in plasmid selective galactose supplemented with 2% raffinose and 50 µM Cu^++^ for 18 hrs at 30 °C with shaking. Cells were scored for autophagy using the *CUP1* controlled GFP-ATG8 reporter by microscopic determination of the location of the fluorescence. (**a**) FM4-64 staining, trypan blue staining and GFP fluorescence of cells expressing GFP-ATG8 are shown in representative cells to illustrate scoring. Cells with accumulation of diffuse fluorescence in the cytoplasm without a concentration of fluorescence in the vacuole, or fluorescence in the cytoplasm in foci even with accumulation in the vacuole, were scored as having delayed or blocked autophagy (-). Cells with fluorescence in the vacuole without punctate cytoplasmic foci, or live cells with no fluorescence, were scored as having autophagy (+). Dead cells were detected with trypan blue staining seen in bright field. (**b**) The fraction of WT, *pbp1*∆, *tip41*∆, or *atg6*∆ cells exhibiting autophagy. The cells shown in Figure 2a are representative examples of what cells looked like when scored as indicated. All types of cells were found in all the strains examined. Still, we have listed the strains the cells shown were found in for completeness. (**c**) The fraction of WT cells exhibiting autophagy and alive with and without overexpression of TDP-43. Cells were grown on selective galactose plates supplemented with 1.6 nM FM4-64, 1% raffinose, and 50 µM Cu^++^ for 18 hrs at 30 °C and scored for autophagy as described in (**a**). The fraction of dead cells was determined as in Figure 1b. Error bars represent the SEM calculated from three independent transformants. About 150-700 cells were scored for each transformant. ** indicates *p* < 0.05 in two-tailed *t*-test.

The level of autophagy was compared in cells with or without overexpression of TDP-43, otherwise grown under identical conditions. Cells deficient in autophagy due to mutation (*atg6Δ*) were used as controls. Autophagy was assayed by observing the breakdown or location of the autophagy reporter, GFP-ATG8, using both and immunoblotting (Figure 1c) and quantitative whole cell imaging (Figure 2a,b).

The level of autophagy in the presence of TDP-43 measured on immunoblots was about 50% of that seen in the absence of TDP-43 overexpression, while *atg6Δ* reduced autophagy to 40% compared to wild-type whether or not TDP-43 was overexpressed (Figure 1c). As expected, addition of the TORC1 inhibitor, rapamycin [33], enhanced autophagy (Figure 1c). Overexpression of TDP-43 had no significant effect on autophagy in the *pbp1Δ* or *tip41Δ* strains (Figure 1c).

For whole cell imaging, GFP-ATG8 fluorescence of viable cells that exhibited diffuse fluorescence in the cytoplasm without a concentration of fluorescence in the vacuole, or fluorescence in the cytoplasm in foci even if accumulation in the vacuole, were scored as having delayed or blocked autophagy. Cells with fluorescence in the vacuole without cytoplasmic punctate foci, or live cells with no fluorescence, were scored as having autophagy (Figure 2a). Using this assay, autophagy was seen in about 85% of wild-type cells without TDP-43 overexpression, 45% of wild-type cells with TDP-43 overexpression, and less than 40% of *atg6Δ* cells with or without TDP-43 overexpression (Figure 2b). Unlike in wild-type cells, TDP-43 did not reduce autophagy in *pbp1Δ* and *tip41Δ* cells (Figure 2b). Overexpression of TIP41 did not affect cell viability whether or not TDP-43 was present (Figure 2c). As reported previously [34] autophagy was enhanced by TIP41 overexpression but we found that overexpression of TDP-43 reduced TIP41 enhanced autophagy (Figure 2c). 

## 4. Discussion

It has long been speculated that autophagy and proteolysis are intimately involved in neurodegenerative diseases. Indeed, a series of experiments show that pathological TDP-43 aggregation alters control of autophagy and that increasing the level of autophagy reduces TDP-43 pathology. For example, several chemical activators of autophagy promote loss of TDP-43 aggregates and reduce TDP-43 associated death of neurons both in vitro and in vivo [23,24,35,36]. Additionally, mutations in genes involved in autophagy such as *UBQLN2, OPTN,* p62, and *VCP,* are linked to ALS [35]. 

A previous report in yeast was inconsistent with the above-mentioned results in neurons, calling into question the idea that increased autophagy is a viable therapeutic approach for TDP-43 toxicity. While this paper [22] primarily examined the effect of the lysosomal pathway on TDP-43 toxicity in yeast, it tangentially reported that TDP-43 enhanced autophagy and that inhibition of autophagy reduced TDP-43 toxicity. Here, using different strains, vectors and growth conditions, we focus on autophagy and TDP-43 toxicity in yeast. Using two distinct assays we find that TDP-43 expression inhibits autophagy. This supports the hypothesis of increased autophagy as a therapeutic.

It has been known for some time that deletion of *PBP1* reduces TDP-43 toxicity in yeast [16]. We show here for the first time that deletion of the related gene, *TIP41,* also rescues TDP-43 toxicity. *PBP1* and *TIP41* are related because deletion of either is lethal in petites and overexpression of either suppresses the petite lethality of mutations reducing import of mitochondrial proteins [20].

Both proteins are involved in autophagy. PBP1 in a liquid-like gel turns on autophagy by inhibiting TORC1 [21,37]. TIP41 activates the SIT4 phosphatase which dephosphorylates TAP42 allowing it to turn on autophagy [34,38]. Different from these papers, we grew cells on synthetic galactose media. Under these conditions we did not detect any effect of deletion of *PBP1* or *TIP41* on autophagy.

Our data suggest that TDP-43 requires both PBP1 and TIP41 for it to inhibit autophagy and be toxic. The mechanism is unknown although their common effect on rescuing petites from inefficient mitochondrial import of proteins may not be the cause since other deletions with that phenotype (*SSB1* or *ICY1*) did not inhibit TDP-43 toxicity. Recalling that, in our conditions, deletion of *PBP1* or *TIP41* only effected autophagy in the presence of TDP-43 overexpression (Figure 2b), we consider the following three hypotheses. One idea is that PBP1 and TIP41 are required for TDP-43 to change into its toxic form which is required for it to inhibit autophagy. Alternatively, PBP1 and TIP41 could encourage cellular processes needed for TDP-43 toxicity and inhibition of autophagy. Finally, TDP-43 together with PBP1 and TIP41 could work through some cellular target to both cause toxicity and inhibit autophagy. 

Here, we speculate on the last possibility, postulating TORC1 as the TDP-43 cellular target that causes toxicity and inhibits autophagy. In this scenario, in the absence of TDP-43, some TORC1 could be inactivated by being sequestered into stress granules or the TOROID structure [39] thereby enhancing autophagy. Expression of TDP-43 could promote the PBP1 and TIP41 dependent release and activation of TORC1, turning autophagy off.

## References

[1] Meriin A.B., Zhang X., He X., Newnam G.P., Chernoff Y.O., Sherman M.Y. (2002). Huntington toxicity in yeast model depends on polyglutamine aggregation mediated by a prion-like protein Rnq1. J. Cell Biol..

[2] Gokhale K.C., Newnam G.P., Sherman M.Y., Chernoff Y.O. (2005). Modulation of prion-dependent polyglutamine aggregation and toxicity by chaperone proteins in the yeast model. J. Biol. Chem..

[3] Johnson B.S., McCaffery J.M., Lindquist S., Gitler A.D. (2008). A yeast TDP-43 proteinopathy model: Exploring the molecular determinants of TDP-43 aggregation and cellular toxicity. Proc. Natl. Acad. Sci. USA.

[4] Ju S., Tardiff D.F., Han H., Divya K., Zhong Q., Maquat L.E., Bosco D.A., Hayward L.J., Brown R.H., Lindquist S. (2011). A yeast model of FUS/TLS-dependent cytotoxicity. PLoS Biol..

[5] Kryndushkin D., Ihrke G., Piermartiri T.C., Shewmaker F. (2012). A yeast model of optineurin proteinopathy reveals a unique aggregation pattern associated with cellular toxicity. Mol. Microbiol..

[6] Kryndushkin D., Wickner R.B., Shewmaker F. (2011). FUS/TLS forms cytoplasmic aggregates, inhibits cell growth and interacts with TDP-43 in a yeast model of amyotrophic lateral sclerosis. Protein Cell.

[7] Fushimi K., Long C., Jayaram N., Chen X., Li L., Wu Y.J. (2011). Expression of human FUS/TLS in yeast leads to protein aggregation and cytotoxicity, recapitulating key features of FUS proteinopathy. Protein Cell.

[8] Barmada S.J., Finkbeiner S. (2010). Pathogenic TARDBP mutations in amyotrophic lateral sclerosis and frontotemporal dementia: Disease-associated pathways. Rev. Neurosci..

[9] Barmada S.J., Skibinski G., Korb E., Rao E.J., Wu J.Y., Finkbeiner S. (2010). Cytoplasmic mislocalization of TDP-43 is toxic to neurons and enhanced by a mutation associated with familial amyotrophic lateral sclerosis. J. Neurosci. Off. J. Soc. Neurosci..

[10] Melamed Z., Lopez-Erauskin J., Baughn M.W., Zhang O., Drenner K., Sun Y., Freyermuth F., McMahon M.A., Beccari M.S., Artates J.W. (2019). Premature polyadenylation-mediated loss of stathmin-2 is a hallmark of TDP-43-dependent neurodegeneration. Nat. Neurosci..

[11] Armakola M., Hart M.P., Gitler A.D. (2011). TDP-43 toxicity in yeast. Methods.

[12] Peggion C., Massimino M.L., Stella R., Bortolotto R., Agostini J., Maldi A., Sartori G., Tonello F., Bertoli A., Lopreiato R. (2021). Nucleolin Rescues TDP-43 Toxicity in Yeast and Human Cell Models. Front. Cell. Neurosci..

[13] Park S.K., Hong J.Y., Arslan F., Kanneganti V., Patel B., Tietsort A., Tank E.M.H., Li X., Barmada S.J., Liebman S.W. (2017). Overexpression of the essential Sis1 chaperone reduces TDP-43 effects on toxicity and proteolysis. PLoS Genet..

[14] Kim J.H., Rahman M.H., Park D., Jo M., Kim H.J., Suk K. (2021). Identification of Genetic Modifiers of TDP-43: Inflammatory Activation of Astrocytes for Neuroinflammation. Cells.

[15] Figley M.D., Gitler A.D. (2013). Yeast genetic screen reveals novel therapeutic strategy for ALS. Rare Dis..

[16] Elden A.C., Kim H.J., Hart M.P., Chen-Plotkin A.S., Johnson B.S., Fang X., Armakola M., Geser F., Greene R., Lu M.M. (2010). Ataxin-2 intermediate-length polyglutamine expansions are associated with increased risk for ALS. Nature.

[17] Sproviero W., Shatunov A., Stahl D., Shoai M., van Rheenen W., Jones A.R., Al-Sarraj S., Andersen P.M., Bonini N.M., Conforti F.L. (2017). ATXN2 trinucleotide repeat length correlates with risk of ALS. Neurobiol. Aging.

[18] Becker L.A., Huang B., Bieri G., Ma R., Knowles D.A., Jafar-Nejad P., Messing J., Kim H.J., Soriano A., Auburger G. (2017). Therapeutic reduction of ataxin-2 extends lifespan and reduces pathology in TDP-43 mice. Nature.

[19] Auburger G., Sen N.E., Meierhofer D., Basak A.N., Gitler A.D. (2017). Efficient Prevention of Neurodegenerative Diseases by Depletion of Starvation Response Factor Ataxin-2. Trends Neurosci..

[20] Dunn C.D., Jensen R.E. (2003). Suppression of a defect in mitochondrial protein import identifies cytosolic proteins required for viability of yeast cells lacking mitochondrial DNA. Genetics.

[21] Yang Y.S., Kato M., Wu X., Litsios A., Sutter B.M., Wang Y., Hsu C.H., Wood N.E., Lemoff A., Mirzaei H. (2019). Yeast Ataxin-2 Forms an Intracellular Condensate Required for the Inhibition of TORC1 Signaling during Respiratory Growth. Cell.

[22] Leibiger C., Deisel J., Aufschnaiter A., Ambros S., Tereshchenko M., Verheijen B.M., Buttner S., Braun R.J. (2018). TDP-43 controls lysosomal pathways thereby determining its own clearance and cytotoxicity. Hum. Mol. Genet..

[23] Barmada S.J., Serio A., Arjun A., Bilican B., Daub A., Ando D.M., Tsvetkov A., Pleiss M., Li X., Peisach D. (2014). Autophagy induction enhances TDP43 turnover and survival in neuronal ALS models. Nat. Chem. Biol..

[24] Wang I.F., Guo B.S., Liu Y.C., Wu C.C., Yang C.H., Tsai K.J., Shen C.K. (2012). Autophagy activators rescue and alleviate pathogenesis of a mouse model with proteinopathies of the TAR DNA-binding protein 43. Proc. Natl. Acad. Sci. USA.

[25] Hayes L.R., Kalab P. (2022). Emerging Therapies and Novel Targets for TDP-43 Proteinopathy in ALS/FTD. Neurother. J. Am. Soc. Exp. Neurother..

[26] Bose J.K., Huang C.C., Shen C.K. (2011). Regulation of autophagy by neuropathological protein TDP-43. J. Biol. Chem..

[27] Chen S., Zhang X., Song L., Le W. (2012). Autophagy dysregulation in amyotrophic lateral sclerosis. Brain Pathol..

[28] Menzies F.M., Fleming A., Caricasole A., Bento C.F., Andrews S.P., Ashkenazi A., Fullgrabe J., Jackson A., Jimenez Sanchez M., Karabiyik C. (2017). Autophagy and Neurodegeneration: Pathogenic Mechanisms and Therapeutic Opportunities. Neuron.

[29] Manogaran A.L., Fajardo V.M., Reid R.J., Rothstein R., Liebman S.W. (2010). Most, but not all, yeast strains in the deletion library contain the [PIN(+)] prion. Yeast.

[30] Park S.K., Arslan F., Kanneganti V., Barmada S.J., Purushothaman P., Verma S.C., Liebman S.W. (2018). Overexpression of a conserved HSP40 chaperone reduces toxicity of several neurodegenerative disease proteins. Prion.

[31] Alberti S., Gitler A.D., Lindquist S. (2007). A suite of Gateway cloning vectors for high-throughput genetic analysis in Saccharomyces cerevisiae. Yeast.

[32] Sherman F.G., Hicks J.B. (1986). Methods in Yeast Genetics.

[33] Yorimitsu T., Zaman S., Broach J.R., Klionsky D.J. (2007). Protein kinase A and Sch9 cooperatively regulate induction of autophagy in Saccharomyces cerevisiae. Mol. Biol. Cell.

[34] Yorimitsu T., He C., Wang K., Klionsky D.J. (2009). Tap42-associated protein phosphatase type 2A negatively regulates induction of autophagy. Autophagy.

[35] Renton A.E., Chio A., Traynor B.J. (2014). State of play in amyotrophic lateral sclerosis genetics. Nat. Neurosci..

[36] Hergesheimer R.C., Chami A.A., de Assis D.R., Vourc’h P., Andres C.R., Corcia P., Lanznaster D., Blasco H. (2019). The debated toxic role of aggregated TDP-43 in amyotrophic lateral sclerosis: A resolution in sight?. Brain A J. Neurol..

[37] Kato M., Yang Y.S., Sutter B.M., Wang Y., McKnight S.L., Tu B.P. (2019). Redox State Controls Phase Separation of the Yeast Ataxin-2 Protein via Reversible Oxidation of Its Methionine-Rich Low-Complexity Domain. Cell.

[38] Jacinto E., Guo B., Arndt K.T., Schmelzle T., Hall M.N. (2001). TIP41 interacts with TAP42 and negatively regulates the TOR signaling pathway. Mol. Cell.

[39] Prouteau M., Desfosses A., Sieben C., Bourgoint C., Lydia Mozaffari N., Demurtas D., Mitra A.K., Guichard P., Manley S., Loewith R. (2017). TORC1 organized in inhibited domains (TOROIDs) regulate TORC1 activity. Nature.

